# The Effect of Non-Solvent Nature on the Rheological Properties of Cellulose Solution in Diluted Ionic Liquid and Performance of Nanofiltration Membranes

**DOI:** 10.3390/ijms24098057

**Published:** 2023-04-29

**Authors:** Sergey O. Ilyin, Anna V. Kostyuk, Tatyana S. Anokhina, Viktoria Y. Melekhina, Danila S. Bakhtin, Sergey V. Antonov, Alexey V. Volkov

**Affiliations:** A.V. Topchiev Institute of Petrochemical Synthesis, Russian Academy of Sciences, 29 Leninsky Prospect, 119991 Moscow, Russia

**Keywords:** cellulose, ionic liquid, polymer solution, phase separation, phase inversion, polymer membrane, nanofiltration, rheology, gelation, laser interferometry

## Abstract

The weak point of ionic liquids is their high viscosity, limiting the maximum polymer concentration in the forming solutions. A low-viscous co-solvent can reduce viscosity, but cellulose has none. This study demonstrates that dimethyl sulfoxide (DMSO), being non-solvent for cellulose, can act as a nominal co-solvent to improve its processing into a nanofiltration membrane by phase inversion. A study of the rheology of cellulose solutions in diluted ionic liquids ([EMIM]Ac, [EMIM]Cl, and [BMIM]Ac) containing up to 75% DMSO showed the possibility of decreasing the viscosity by up to 50 times while keeping the same cellulose concentration. Surprisingly, typical cellulose non-solvents (water, methanol, ethanol, and isopropanol) behave similarly, reducing the viscosity at low doses but causing structuring of the cellulose solution and its phase separation at high concentrations. According to laser interferometry, the nature of these non-solvents affects the mass transfer direction relative to the forming membrane and the substance interdiffusion rate, which increases by four-fold when passing from isopropanol to methanol or water. Examination of the nanofiltration characteristics of the obtained membranes showed that the dilution of ionic liquid enhances the rejection without changing the permeability, while the transition to alcohols increases the permeability while maintaining the rejection.

## 1. Introduction

Due to its chemical resistance, cellulose is a promising material in the field of membrane technology. The history of the use of cellulose is long and spans decades: as early as 1944, W.J. Kolff used a cellulose film called cellophane for the first time in a dialysis machine for blood purification [1]. Eventually, membranes based on cellulose and its derivatives have found wide applications for water treatment and filtration [2]. As a membrane material, cellulose is interesting for use in the nanofiltration of organic media, as it does not dissolve in organic solvents, including high-polar aprotic ones. However, the chemical resistance of cellulose is also a disadvantage. The formation of membranes from cellulose is challenging due to a limited number of solvents for its processing [3,4,5,6]. Most of the cellulose solvents are two- and three-component systems, such as dimethylacetamide/LiCl [7,8], *N*-methyl-2-pyrrolidone/LiCl [9], dimethyl sulfoxide/paraformaldehyde [10,11], aqueous solutions of H_3_PO_4_ [12], and so on. The multi-component composition of these solvents leads to the complexity of dissolution, their instability, and frequently high toxicity.

Among single-component solvents, only *N*-oxides were known for a long time [13], and *N*-methylmorpholine *N*-oxide (NMMO) was the most common. The advantages of this solvent are the safety for the environment and humans and the ease of processing cellulose after its dissolution. The environmentally friendly NMMO process is used in industry to produce cellulose fibers and yarns, which has created enormous competition for the viscose method [14,15,16,17,18,19]. The literature widely describes the use of NMMO for shaping cellulose membranes for gas separation [20,21], pervaporation [22], hemodialysis [23], ultra- [24,25,26], and nanofiltration [27]. The main disadvantage of NMMO as a solvent is its crystalline state at room temperature. Therefore, the dissolution must be at high temperatures exceeding 100 °C [18,20,22,26,28,29,30,31,32] or using a co-solvent [33]. During the formation of membranes, it is necessary to carefully control the temperature regime, as a decrease in temperature can lead to undesirable crystallization of the forming solution, which will not allow obtaining a membrane with the required structure.

In recent years, ionic liquids (ILs) have become of great interest as single-component solvents for cellulose. They are a class of organic salts whose melting point is usually below 100 °C [34]. Although the dissolving ability of organic salts to cellulose was discovered almost simultaneously with that of *N*-oxides—in the 1930s [35], this discovery did not attract attention for a long time until the founding of salts in the liquid state at room temperature. There are many studies on the dissolution of cellulose in various ILs [36,37,38,39,40]. Ionic liquids have several advantages: their properties can be adjusted by changing the cation or anion [41,42], they are non-toxic and environmentally safe due to their absolute non-volatile nature, and they can dissolve cellulose with a very high degree of polymerization [43]. However, the main advantage of ILs compared to NMMO is that the dissolution of cellulose and subsequent processing of its solution can be at temperatures below 100 °C. The possibility of modifying the properties of an IL by changing the nature of its cation or anion also alters the dissolving ability, e.g., anions, such as phosphates and phosphonates, allow for dissolving cellulose at room temperature [44,45].

Cellulose solutions in ILs are considered frequently from the viewpoint of forming membranes from them. The literature describes works using ionic liquids for producing ultra- [46] and nanofiltration membranes [47]. However, the disadvantage of ionic liquids is their high viscosity due to high-energic interionic interactions. Since the viscosity of a polymer solution is proportional in the first approximation to that of the used solvent, the high viscosity of the latter limits the maximum content of cellulose in the forming solution. In turn, it can restrict the density of the produced membranes. To dilute the cellulose solution and make a fine-pore membrane, a co-solvent with low viscosity can be additionally used with ionic liquid, e.g., acetone [48] or dimethyl sulfoxide (DMSO) [49,50,51,52].

Cellulose is of interest as a membrane material for the nanofiltration of organic media. The operating pressure during nanofiltration is 2–3 MPa, and the particle size of the extracted target components is in the range of molecular weights from 200 to 1400 g/mol [53]. The main requirements for these membranes are mechanical strength, chemical resistance in organic media, high productivity, and good selectivity in separation processes. In addition, the biorenewability and biodegradability of the membrane material are distinct modern requirements in present times. For these reasons, cellulose is a natural biodegradable material that can provide nanofiltration membranes with exceptionally high mechanical and chemical stability. However, the performance and selectivity of membranes depend not only on the membrane material but also on the method and parameters of its formation, which determine the presence of a specific structure that should provide high performance and selectivity.

In practice, nanofiltration mainly uses either uniformly porous membranes with a pore size less than 2 nm [54,55] or asymmetric membranes consisting of an upper dense selective layer and a porous sublayer of the same chemical composition. These membranes result from the so-called phase inversion method [56,57]. The initial solution consisting of only one phase is forced to disintegrate into two: a polymeric, being a solid phase that forms the membrane matrix, and a liquid phase, originating from the membrane pores [58]. There are several different approaches to implementing the phase inversion method [55,57,59,60], but nanofiltration membranes most commonly are obtained by immersing a polymer solution into a coagulation bath with a non-solvent (a precipitant) [55]. In this case, two possible variants of membrane formation can exist. In the first case, the diffusion rate of the precipitant into the polymer solution is much faster than that of the solvent into the precipitant medium, or the diffusion rates are comparable. This situation leads to instant coagulation of the polymer when its solution is immersed in the coagulation bath. As a result, asymmetric membranes with a dense selective layer and an underlying layer with finger-like pores are created. If the diffusion rate of the solvent exceeds that of the precipitant, polymer deposition is slow. After immersion in the coagulation bath, the solid phase is produced after some time. With such a slow exchange of solvent and non-solvent, a dense porous structure is formed within the membranes, similar to a sponge [60]. Thus, to prepare a membrane with a specific structure and morphology, it is necessary to control the diffusion rate of the solvent and non-solvent.

The polymer concentration in the solution can be varied to control the diffusion rate. This approach will increase or decrease the viscosity of the solution and allow the formation of a denser or more porous membrane structure. However, it is necessary to choose a suitable solvent for a polymer. The polymer must be soluble in the selected solvent, and the solvent must be well miscible with a non-solvent. In the case of cellulose, the choice of solvent is limited, but that of possible co-solvents is wide when it will be paired with an ionic liquid. In addition, the selection of non-solvent plays an essential role in the phase inversion process [55,60]. Precipitants used in pure form are called hard, while applied as a mixture of a non-solvent with a solvent are soft. Hard precipitants cause a strong supersaturation of the polymer solution, which leads to the formation of a myriad of new-phase nuclei and their rapid growth to a finely porous membrane [61]. In addition, air humidity, non-solvent temperature, time from shaping to coagulation, and the duration of the coagulation itself are also essential parameters in creating nanofiltration membranes by the phase inversion method [62,63]. For example, an increase in temperature promotes the formation of membranes characterized by lower permeability but higher selectivity [55].

The process of forming membranes from cellulose solutions in NMMO is well studied. Some works examine the effect of non-solvents, such as aliphatic alcohols [27,28,64], aqueous solutions of NMMO [18,23,26,65], and viscous glycols [66], on the morphology, structure, and filtration characteristics of membranes from solutions in NMMO. However, the influence of the non-solvent nature on the structure formation of nanofiltration membranes from cellulose solutions in ionic liquids containing co-solvents has not yet been studied. This work aimed to investigate the effect of different non-solvents on the morphology and nanofiltration characteristics of membranes obtained from cellulose solutions in an ionic liquid containing DMSO as a co-solvent. DMSO is a non-toxic alternative solvent for making polymer membranes [67,68,69,70], and therefore its use in a mixture with ionic liquid as another green solvent is ecologically reasonable. The study is structured as follows: first, we will find the optimal ratio between DMSO and one of three common ionic liquids to obtain a moderately viscous cellulose solution, and then we will consider the effect of the nature of a non-solvent on cellulose coagulation and membrane properties.

## 2. Results and Discussion

### 2.1. Rheology of Cellulose Solutions in Diluted Ionic Liquids

The weak point of ionic liquids is their high viscosity, which increases the viscosity of cellulose-containing forming solutions, limits the mass fraction of cellulose in them, and thus may reduce the performance of formed cellulose films and fibers. It is reasonable to reduce the viscosity of cellulose solutions by adding a low-viscosity co-solvent, but cellulose has no such ability because of poor solubility. Therefore, a low-viscous non-solvent, which has a low potential to deteriorate the dissolution ability of ionic liquid, remains to be used as a nominal co-solvent. DMSO can be this nominal co-solvent, as it does not dissolve cellulose but is aprotic and has a very high polarity, i.e., it should weakly impair ionic liquid. However, it is unclear which ionic liquid will best retain its dissolving ability for cellulose in the presence of DMSO, how greatly the viscosity of the cellulose solution will be reduced, and how much DMSO can be added without the loss of cellulose solubility.

For testing, we used three ionic liquids most commonly used to dissolve cellulose, which contained 25, 50, or 75 wt% DMSO. The mass fraction of cellulose in all the solutions under consideration was 14 wt% without exception, as preliminary tests showed that this concentration allowed obtaining cellulose films from cellulose solutions in all three ionic liquids in their pure form. The solutions were examined at 25 °C, whereas their preparation was carried out at 80 °C since cellulose dissolves for an infinitely long time at 25 °C. For assessing the quality of cellulose solutions, we measured the dependence of their viscosity (*η*) on the shear stress (*σ*) and that of their storage (*G*′) and loss (*G*″) moduli on the angular frequency (*ω*), as this allows for determining the gel formation due to a decrease in the solubility of a polymer [71,72].

**Figure 1:**
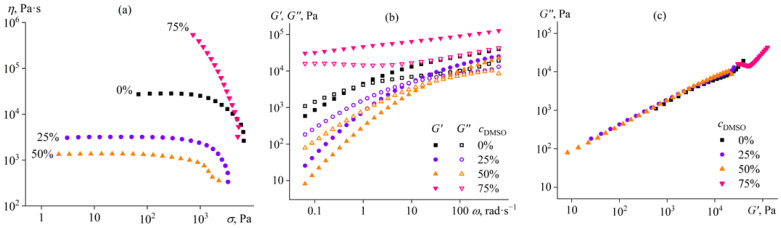
Dependences of (**a**) viscosity on shear stress, (**b**) storage and loss moduli on angular frequency, and (**c**) Cole–Cole plots for 14% cellulose solutions in [EMIM]Ac/DMSO mixture at 25 °C. The mass fraction of DMSO in the complex solvent is given near the curves or in the legend.

The cellulose solution in 1-ethyl-3-methylimidazolium acetate ([EMIM]Ac) is a non-Newtonian fluid; its viscosity is constant at low shear stresses but decreases at high ones (Figure 1a). The cellulose, with a given polymerization degree, forms an entangled solution in this ionic liquid at the used concentration [73,74]. Thus, the decrease in viscosity results from a reduction in the density of macromolecular entanglements at high shear rates due to stretching, orientation, and disentanglement of macromolecular chains [75]. The addition of up to 50% DMSO to the cellulose solution in the ionic liquid (while maintaining the same cellulose concentration) reduces its viscosity. This reduction is due to the lower viscosity of DMSO (2 mPa·s) compared to [EMIM]Ac (26 mPa·s). However, when a complex solvent composition is used with 75% DMSO, the viscosity of the cellulose solution sharply increases, and the solution starts to exhibit a strongly non-Newtonian behavior. It has previously been shown that adding up to 50% DMSO to a cellulose solution in ionic liquid reduces its viscosity without changing the relaxation process [76], but higher concentrations of DMSO were not considered, and no increase in the viscosity of the cellulose solution was found after diluting the ionic liquid.

The explanation for the high viscosity of the composition containing 75% DMSO in the complex solvent is its gelation; the storage modulus of this sample weakly depends on the angular frequency and exceeds the loss modulus (Figure 1b). In contrast, the systems with lower DMSO content are typical polymer solutions; at low frequencies, their loss modulus exceeds the storage modulus, *G*′~*ω*^2^, and *G*″~*ω*. Gel formation is because DMSO is not a solvent for cellulose, unlike [EMIM]Ac. Miscibility of compounds generally improves at higher temperatures, and cellulose is soluble in a mixture of 75% DMSO and 25% [EMIM]Ac at the mixing temperature of 80 °C. However, the complete solubility disappears as the temperature decreases, but the resulting phase separation occurs infinitely slowly due to tremendous increases in viscosity since the cellulose-rich phase becomes a continuous medium [77]. In our case, if we consider the interdependences of storage and loss moduli in Cole–Cole coordinates, it appears that the experimental points for all samples lie approximately on the same straight line (Figure 1c). The invariance of the Cole–Cole plots means that DMSO does not change the microstructure of the cellulose solution [78,79], whose gelation is due to glass transition rather than phase separation, which is prevented by an extremely high viscosity thanks to the glass state [80].

**Figure 2:**
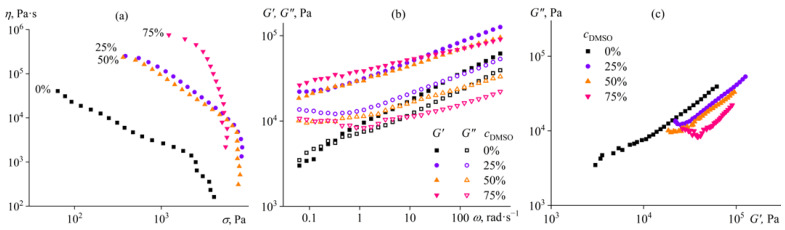
Dependences of (**a**) viscosity on shear stress, (**b**) storage and loss moduli on angular frequency, and (**c**) Cole–Cole plots for 14% cellulose solutions in [EMIM]Cl/DMSO mixture at 25 °C. The mass fraction of DMSO in the complex solvent is given near the curves or in the legend.

A cellulose solution in pure 1-ethyl-3-methylimidazolium chloride ([EMIM]Cl) is a non-Newtonian fluid whose viscosity decreases with an increase in shear stress, even low ones (Figure 2a). In other words, shear-thinning behavior is caused not only by the disentanglement of macromolecules but also by the destruction of some structures, possibly macromolecular associates that break at lower shear stresses than the disentanglement of macromolecules [81,82]. In addition, [EMIM]Cl is in a crystalline state at 25 °C. The introduction of cellulose into the ionic liquid suppresses its crystallization due to the formation of hydrogen bonds between macromolecules and solvent anions. Nevertheless, some solvent ions may not be associated with cellulose and form local crystallites that act as structural nodes in the network of macromolecular entanglements. These nodes may also break when applying shear stress, decreasing viscosity.

When 25% or 50% of DMSO is added to the solvent composition, the viscosity of the solution increases by a decimal order of magnitude without a change in non-Newtonian behavior (Figure 2a). The addition of more DMSO, up to 75%, leads to further growth in viscosity and more substantial shear-thinning behavior. Deterioration of solvent quality may cause a more significant association of macromolecules but should not enhance the crystallization of the ionic liquid (it should instead inhibit it due to a decrease in its concentration). On this basis, the increase in viscosity in the presence of DMSO indicates a greater probability of structure formation due to the association of macromolecules rather than the local crystallization of the ionic liquid. Furthermore, the frequency dependences of storage and loss moduli are atypical for ordinary polymer solutions when *G*′~*ω* and *G*″~*ω*^2^ at *ω* → 0. For the sample without DMSO, both moduli have similar values and decline with a decrease in angular frequency with the same slope of 0.3 at low frequencies (Figure 2b). The addition of DMSO enhances the structural formation of the solution; the storage modulus becomes higher and less dependent on frequency, significantly exceeding the loss modulus. Moreover, the microstructure of the system changes with each addition of DMSO since the Cole–Cole plots do not overlap with each other (Figure 2c), unlike the case of using [EMIM]Ac (see Figure 1c). DMSO is not a solvent for cellulose, making the solution a gel due to stronger macromolecular association even at its 25% mass fraction, and each addition of DMSO increases the gelling of the system and its heterogeneity. Thus, dilution of [EMIM]Cl with DMSO is not a way to reduce the viscosity of the cellulose solution to facilitate its shaping.

**Figure 3:**
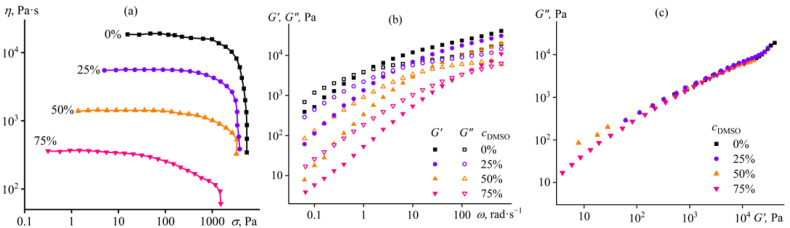
Dependences of (**a**) viscosity on shear stress, (**b**) storage and loss moduli on angular frequency, and (**c**) Cole–Cole plots for 14% cellulose solutions in [BMIM]Ac/DMSO mixture at 25 °C. The mass fraction of DMSO in the complex solvent is given near the curves or in the legend.

The solution of cellulose in 1-butyl-3-methylimidazolium acetate ([BMIM]Ac) is similar in non-Newtonian behavior to that in [EMIM]Ac (Figure 3a). Meanwhile, the solution in [BMIM]Ac has a lower viscosity than in [EMIM]Ac (18,400 Pa·s versus 28,300 Pa·s), although the viscosity of pure [EMIM]Ac is lower when compared with [BMIM]Ac (26 mPa·s versus 118 mPa·s). This means that [EMIM]Ac is the thermodynamically better solvent for cellulose, as macromolecular coils expand more strongly in better solvents and have a higher intrinsic viscosity, resulting in more viscous dilute and concentrated solutions [83]. The addition of DMSO gradually and significantly reduces the viscosity of the solution, and even 75% DMSO does not cause gelation. In this respect, DMSO less significantly impairs the dissolving ability of [BMIM]Ac if compared to [EMIM]Ac, where the addition of 75% DMSO turns the cellulose solution into a gel (see Figure 1). The absence of gelation is also confirmed by frequency dependences of storage and loss moduli (Figure 3b), which are typical for an ordinary concentrated polymer solution since *G*′~*ω* and *G*″~*ω*^2^ are in the terminal zone even at 75% DMSO mass fraction in the complex solvent. The close overlapping of the Cole–Cole plots also confirms the invariability of the solution microstructure in the presence of DMSO (Figure 3c), although a slight deviation of the dependence for the most DMSO-rich system hints at a possible beginning of a structural transformation.

Thus, dilution of [EMIM]Ac or [BMIM]Ac with DMSO reduces the viscosity of the cellulose solution. Mainly, the mechanism of viscosity reduction involves a decrease in the viscosity of the complex solvent (*η*_0_) due to the lower viscosity of DMSO compared to that of the ionic liquids. Additionally, the viscosity of polymer solutions may decline due to the decrease in the thermodynamic quality of the solvent, leading to the contraction of macromolecular coils and a reduction in their volume. However, the deterioration of the solvent quality can lead to the gelation of the solution, which occurs at 75% DMSO content in [EMIM]Ac. Meanwhile, cellulose solutions in [EMIM]Cl are in an associated state with high viscosity, which DMSO only increases. Based on this, only [EMIM]Ac and [BMIM]Ac are suitable for dilution with DMSO. [BMIM]Ac is preferable, as it allows for deeper dilution. However, the forming solution should not be too low-viscous, as it will easily flow out during membrane shaping. A viscosity of approximately 1000 Pa·s is optimal for forming, neither too high nor too low. Therefore, the ratio of [BMIM]Ac/DMSO = 25/75 is unsuitable for preparing a 14% cellulose shaping solution due to its low viscosity, although this ratio potentially allows for obtaining more concentrated cellulose solutions for their shaping. Comparing 14% cellulose solutions in acetate ionic liquids diluted with 50% DMSO, the viscosity of the solution in [EMIM]Ac is lower than in [BMIM]Ac (1370 Pa·s versus 1430 Pa·s). In pure ionic liquids, the solution in [EMIM]Ac had higher viscosity at the same time (28,300 Pa·s versus 18,400 Pa·s). This seeming inconsistency confirms that DMSO significantly degrades the solvent quality in the case of [EMIM]Ac, resulting in a more substantial decrease in the viscosity of the cellulose solution due to the contraction of macromolecular coils.

**Figure 4:**
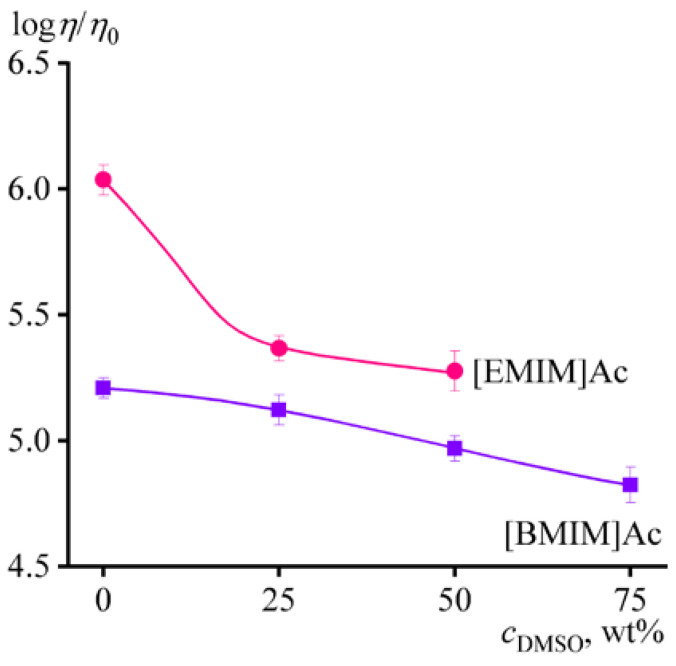
Dependences of the relative viscosity of 14% cellulose solution on the mass fraction of DMSO in ionic liquid.

An additional indication of the deterioration in the solvent quality and consequent conformational changes of macromolecules is the dependence of the relative viscosity of 14% cellulose solution on the content of DMSO in the complex solvent (Figure 4). If macromolecules did not change their conformation, the solution/solvent viscosity ratio (*η*/*η*_0_) would be a constant value. In our case, relative viscosity drops with the dilution of ionic liquids, indicating a decrease in the size of macromolecular coils. Pure [EMIM]Ac is the thermodynamically best solvent since the relative viscosity of the cellulose solution is higher. However, the quality of this ionic liquid deteriorates sharply upon the addition of DMSO but still remains higher than that of [BMIM]Ac containing the same amount of DMSO. Thus, a 50% solution of [EMIM]Ac in DMSO is the better cellulose solvent; moreover, the viscosity of 14% cellulose solution in it is lower compared to using similarly diluted [BMIM]Ac, which results from the lower viscosity of pure [EMIM]Ac. For these reasons, 14% cellulose solutions in pure [EMIM]Ac and equimass [EMIM]Ac/DMSO mixtures were bases for further experiments.

### 2.2. Effect of Non-Solvent Addition on the Rheological Properties of Cellulose Solutions

Since DMSO impairs the dissolving ability of ionic liquid, even a low quantity of a non-solvent can result in phase separation of a cellulose solution in a diluted ionic liquid. For this reason, the effect of non-solvent on the rheological properties of a cellulose solution and indirectly its phase state was studied in the absence of DMSO to have a possibility for varying the mass fraction of non-solvent more significantly and also to avoid a specific DMSO/non-solvent interaction. Water, methanol, ethanol, and isopropanol were used as non-solvents, assuming a decrease in the ability to be a proton donor (i.e., to initiate hydrogen bonds) in the designated series (the Kamlet–Taft solvatochromic parameter *α* that characterizes H-bond donation is 1.17, 0.98, 0.86, and 0.76, respectively [84,85]).

**Figure 5:**
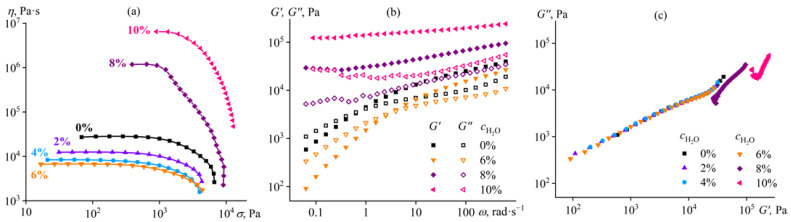
Dependences of (**a**) viscosity on shear stress, (**b**) storage and loss moduli on angular frequency, and (**c**) Cole–Cole plots for 14% cellulose solutions in [EMIM]Ac/water mixture at 25 °C. The water mass fraction in [EMIM]Ac is given near the curves or in the legend.

The addition of small amounts of water, not exceeding 6%, to 14% cellulose solution in [EMIM]Ac results in a gradual reduction in its viscosity, as shown in Figure 5a. In other words, water acts as a nominal co-solvent at low concentrations, similar to DMSO. The reduction in the solution’s viscosity occurs both due to the decrease in the viscosity of the complex solvent, as water has a lower viscosity than the ionic liquid, and thanks to the deterioration of the solvent’s thermodynamic quality, leading to a decrease in the size of macromolecular coils. However, the addition of 8–10% water causes a sharp rise in viscosity and strengthening of non-Newtonian behavior, which may indicate gel formation.

Measurement of frequency dependences of storage and loss moduli confirms the assumption of gel formation: *G*′ > *G*″ and *G*′ ≈ *const* at a water mass fraction of 8–10% (Figure 5b). At the same time, the frequency dependences of the moduli at a water content of 6% are similar to that of a water-free solution; there is a terminal zone in the low-frequency region that transitions to a nominal rubbery plateau region with an increase in angular frequency. The similarity of the curves indicates that the gradual addition of water does not cause any new interactions between cellulose macromolecules until they lose solubility and gel formation occurs. Cole–Cole plots confirm this conclusion (Figure 5c). Experimental points overlay each other at water contents from 0% to 6%, indicating the invariance of the microstructure. An addition of 8% water triggers the formation of a new microstructure, while an increase in the water concentration to 10% alters this structure, strengthening the gel formation.

It should be noted that the mechanisms of gel formation in a cellulose solution in [EMIM]Ac under the influence of water and DMSO differ, despite the apparent similarity of the rheological curves (Figure 1a and Figure 5a, Figure 1b and Figure 5b). In the case of DMSO, gel formation occurs due to the glass transition of the cellulose solution, whose very high viscosity prevents phase separation (the Cole–Cole plots are almost on the same line, see Figure 1c). In contrast, water causes gel formation through the microphase separation (the Cole–Cole dependences shift, indicating a change in microstructure, see Figure 5c) and the subsequent glass transition of the polymer-enriched continuous phase. In this respect, the action of water is similar to that of DMSO on the cellulose solution in [EMIM]Cl, where each dose of DMSO enhanced gel formation by changing the microstructure of the gel (see Figure 2c).

**Figure 6:**
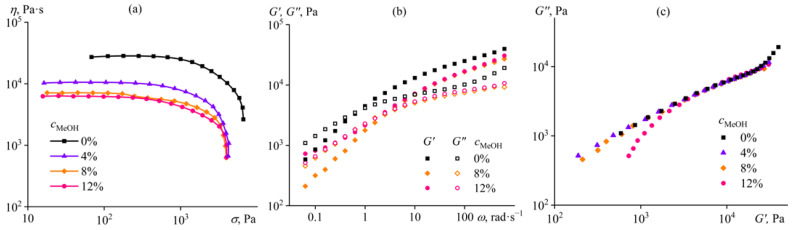
Dependences of (**a**) viscosity on shear stress, (**b**) storage and loss moduli on angular frequency, and (**c**) Cole–Cole plots for 14% cellulose solutions in [EMIM]Ac/methanol mixture at 25 °C. The methanol mass fraction in [EMIM]Ac is given near the curves or in the legend.

**Figure 7:**
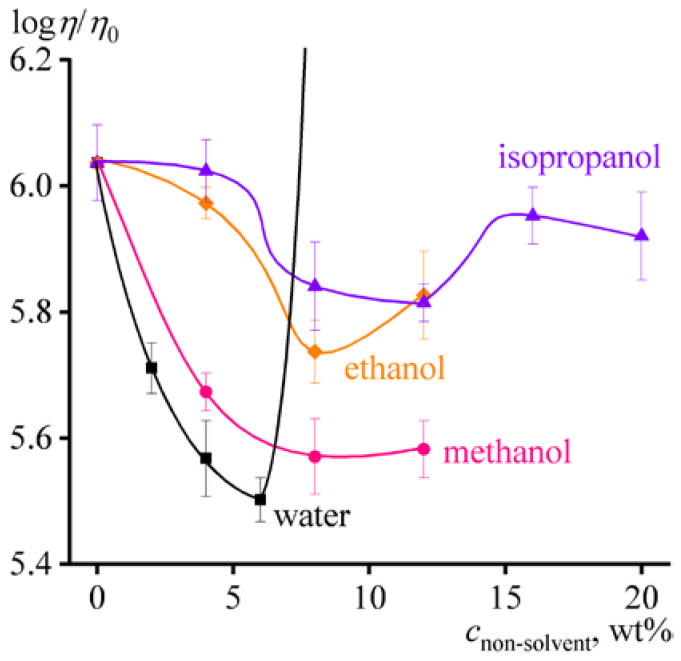
Dependences of the relative viscosity of 14% cellulose solution on the mass fraction of non-solvent in [EMIM]Ac.

Additions of methanol (MeOH) to cellulose/[EMIM]Ac solution in comparable amounts of up to 12% do not result in gel formation. The solution’s viscosity decreases with increasing mass fraction of methanol without changing the flow behavior (Figure 6a). The first 4% portion of methanol reduces the viscosity more intensively than the subsequent second 4% dose, whereas the third dose only slightly lowers the viscosity. A similar situation occurred when adding water, where the effect of viscosity reduction decreased with each subsequent portion of water (see Figure 5a). Since the cellulose solution’s viscosity falls due to a dilution of ionic solvent (which occurs monotonically with each dose) and a deterioration in its thermodynamic quality, the gradually waning effect of viscosity reduction points to the sharp decline in the ionic liquid’s thermodynamic quality at low non-solvent contents. This conclusion also results from the dependences of the relative viscosity of the cellulose solution on the non-solvents’ mass fraction in the complex solvent (Figure 7); the relative viscosity, which depends on the thermodynamic quality of the solvent but not its dynamic viscosity, rapidly decreases even at low water or methanol concentrations. At the same time, the relative viscosity sharply rises due to gelation at elevated water mass fractions but reaches a constant level when methanol is in use. One would expect the rise of relative viscosity at higher concentrations of methanol, which would induce gelation. Nevertheless, the less intensive reduction in relative viscosity and absence of gel formation at comparable concentrations indicate that methanol is a better nominal co-solvent for cellulose than water, which is a harder non-solvent.

Methanol up to a mass fraction of 8% does not change the pattern of frequency dependences of the storage and loss moduli of the cellulose solution, which are typical for an ordinary concentrated polymer solution (Figure 6b). However, the change occurs at a methanol content of 12%, the storage modulus tends to come to a constant value and exceeds the loss modulus in the low-frequency region, which may indicate a structured state of the solution [86]. Indeed, the Cole–Cole plots show the difference between the experimental points and, thus, the different microstructure of the solution containing 12% methanol in [EMIM]Ac (Figure 6c). Similar rheological behavior, on the background of other samples, was for the cellulose solution in [BMIM]Ac containing as much as 75% DMSO (see Figure 3c). It turns out that at a certain content of non-solvent, the cellulose solution enters a weakly structured pre-gel state, which practically does not manifest itself in rheological terms and is detected only as a low-frequency anomaly of the storage modulus or in a comparative analysis of Cole–Cole plots.

**Figure 8:**
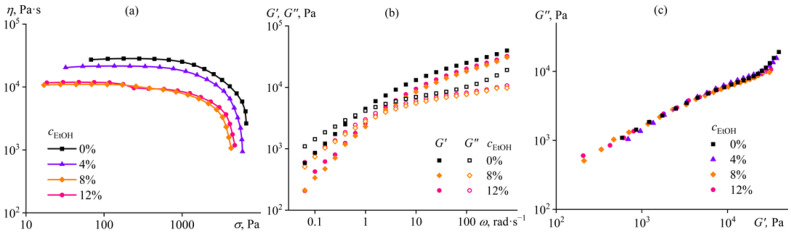
Dependences of (**a**) viscosity on shear stress, (**b**) storage and loss moduli on angular frequency, and (**c**) Cole–Cole plots for 14% cellulose solutions in [EMIM]Ac/ethanol mixture at 25 °C. The ethanol mass fraction in [EMIM]Ac is given near the curves or in the legend.

The behavior of the cellulose solution upon adding ethanol (EtOH) to the ionic liquid is almost the same as when using methanol. In the same mass fractions, ethanol reduces the viscosity of the cellulose solution with decreasing efficiency but does not cause gelation (Figure 8a). Ethanol similarly lowers storage and loss moduli but without changing the pattern of their frequency dependences, even at its 12% content (Figure 8b). In this respect, 12% ethanol does not induce weak structuring, which also results from the invariance of the Cole–Cole plots (Figure 8c). Thus, ethanol is a better nominal co-solvent than methanol, and the less intense decrease in the relative viscosity of the cellulose solution (less deterioration of the solvent quality) also supports this conclusion (Figure 7).

**Figure 9:**
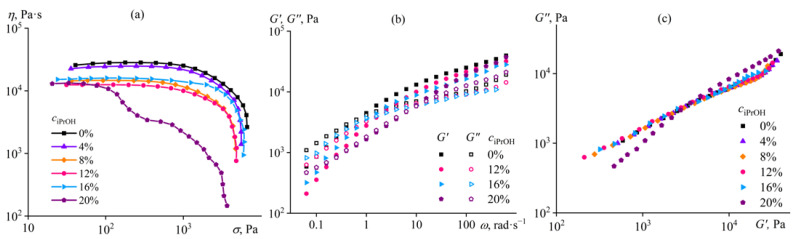
Dependences of (**a**) viscosity on shear stress, (**b**) storage and loss moduli on angular frequency, and (**c**) Cole–Cole plots for 14% cellulose solutions in [EMIM]Ac/isopropanol mixture at 25 °C. The isopropanol mass fraction in [EMIM]Ac is given near the curves or in the legend.

Since in the sequence water, methanol, and ethanol, there is an improvement in the quality of these liquids as nominal cellulose co-solvents, we can expect that isopropanol will deteriorate the dissolution potential of the ionic liquid even more weakly and hence less strongly contribute to the phase separation of the cellulose solution. For this reason, the mass fraction of isopropanol (iPrOH) in [EMIM]Ac has been varied within a broader range, up to 20%. Indeed, the addition of up to 12% isopropanol does not induce gelation and only reduces the viscosity of the cellulose solution (Figure 9a) with a decrease in its storage and loss moduli (Figure 9b). The introduction of 16% isopropanol leads to an unexpected growth in viscosity relative to the value observed when using 12% of this alcohol. In this case, the storage and loss moduli increase in the low-frequency region but decrease in the high-frequency one. We can assume that weak specific interactions between cellulose macromolecules start to appear at 16% isopropanol content, not generating a network (since *G*′~*ω*^2^ at low frequencies) due to the small number of bonds but raising the viscosity. A higher isopropanol content equal to 20% intensifies the weak intermolecular interactions, i.e., increases their density, which results in the formation of a loose network; the low-frequency storage and loss moduli become comparable and less frequency-dependent (Figure 9b). If we examine the Cole–Cole plots (Figure 9c), the presence of a specific structure in the cellulose solution in the [EMIM]Ac/iPrOH mixture with a ratio of 80/20 is evident, as the experimental points for it do not coincide with those for the other solutions, both in the low and high modulus region. In contrast, the Cole–Cole plot shows only a very slight deviation in the area of high moduli when the isopropanol content is 16%, i.e., there is almost no change in the microstructure of the cellulose solution at this non-solvent mass fraction.

Thus, 20% isopropanol in the ionic liquid initiates a weak structuring of the cellulose solution, similar to 12% methanol. In other words, isopropanol is an even better nominal co-solvent for cellulose (or its softer non-solvent) than other alcohols. This conclusion is confirmed by the behavior of the relative viscosity, which decreases the least when isopropanol is in use (Figure 7). In this case, while the decline in relative viscosity is associated with the deterioration of the thermodynamic quality of the solvent, the passage to its growth is due to the start of weak structuring of cellulose macromolecules. At the same time, the transition from isopropanol through ethanol and methanol to water not only intensifies the relative viscosity drop but also shifts the starting point of cellulose solution structuring toward lower non-solvent concentrations. Under this interpretation of structural changes in the cellulose solution, the essential difference between the actions of water and alcohols is that water causes strong structuring rather than weak. Structuring by water is expressed in gel formation (see Figure 5a,b), while alcohols cause an anomalous dependence of storage and loss moduli at low frequencies. Meanwhile, the flow curve of the cellulose solution in [EMIM]Ac/iPrOH mixture with an 80/20 ratio is quite specific, as its viscosity drops sharply even at moderate shear stresses exceeding 100 Pa (Figure 9a). This drop in viscosity can be attributed to the phase separation of the solution, initiated by the action of shear due to the stretching of macromolecular chains and a reduction in the entropy of the system [87,88,89]. Due to the phase separation, there is a transition from the flow of the solution to the wall slip of the resulting decomposed system over a layer of the released solvent, accompanied by a fall in the measured effective viscosity.

In summary, all of the considered non-solvents act as nominal co-solvents of cellulose at low concentrations, reducing the viscosity of its solution. The decrease in viscosity is due both to the lower viscosities of non-solvents (0.54–2 mPa·s) relative to [EMIM]Ac (26 mPa·s) and to the deterioration of thermodynamic affinity to cellulose and folding of macromolecules, expressed as a decline in the relative viscosity of their solution. However, at some contents of non-solvents, increasing in the row of water, methanol, ethanol, and isopropanol, the structuring of the solution happens. Water causes strong structuring with gel formation, while alcohols weakly structure the cellulose solution, almost without changing its rheological properties. Meanwhile, a further increase in the content of any non-solvent should cause the transition from weak or strong structuring to phase separation of the cellulose solution. The effect of high concentrations of non-solvent on the phase state of the cellulose solution cannot be investigated by rheometry because of the wall slip of phase-separated samples, but other methods still may help, e.g., laser interferometry.

### 2.3. Phase Separation and Diffusion in Contact of Cellulose Solution with Non-Solvents

**Figure 10:**
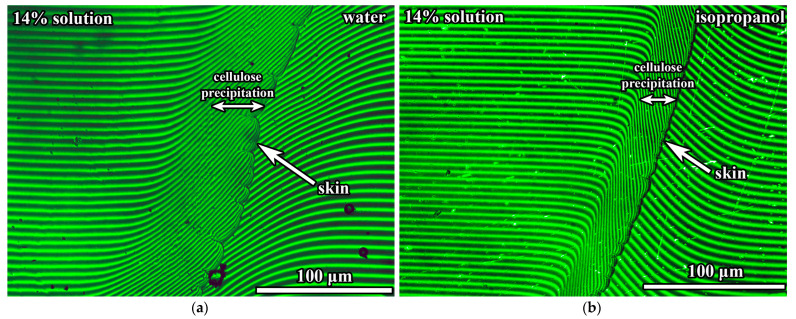
Interferograms of mutual diffusion zones of 14% cellulose solution in [EMIM]Ac/DMSO (50/50) brought into contact with a non-solvent at 25 °C after 90 s.

Laser interferometry allows for investigating the phase separation when a polymer solution comes into contact with a non-solvent in a narrow gap between semi-transparent glass plates [27,66]. Typical interferograms, visually identical for all studied systems, are presented in Figure 10. A pure cellulose solution is located on the left side of the interferograms, while a pure non-solvent is on the right side. When in contact, they form a diffusion zone that results in curved interference fringes due to the different refractive indices of the cellulose solution, non-solvent, and their mixtures, in which the concentration of non-solvent changes from 0% to 100%. The precipitation of cellulose films happens in the diffusion zone, but only one distinct phase boundary is visible in the area enriched with the non-solvent for all systems, representing the membrane skin (indicated by the arrow in Figure 10). In the cellulose-rich area, there is no clear border of cellulose film, pointing to its low-dense structure and a smooth concentration transition from the precipitated cellulose to its solution. In all cases, the membranes are optically transparent, indicating that they do not contain macro-pores, at least the ones larger than half of the wavelength of the used green laser (i.e., greater than 266 nm).

**Figure 11:**
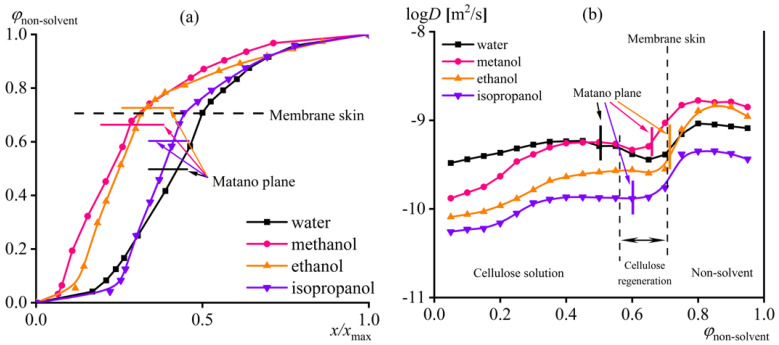
The concentration distribution of the non-solvent along the normalized length of the diffusion zone (**a**) and the dependences of the solvent/non-solvent interdiffusion coefficient on the volume fraction of the non-solvent (**b**) when it comes into contact with 14% cellulose solution in [EMIM]Ac/DMSO (50/50) at 25 °C.

Mutual diffusion of substances occurs during membrane formation: the solvents transit from the cellulose solution into the non-solvent medium and vice versa. The rate of mutual diffusion can be estimated based on the analysis of interferograms after a known time from the beginning of the experiment, which also allows for determining the concentration of the non-solvent causing phase separation (i.e., skin formation). In the first step, interferograms allow for calculating the change in the volume fraction of non-solvent (*φ*_non-solvent_) along the diffusion zone (Figure 11a). Since the rate of mutual diffusion differs when using different non-solvents, this leads to obtaining diffusion zones of different lengths (*x*_max_) at an equal time from the beginning of contact. For instance, the diffusion zone is grander when using water (Figure 10a) than isopropanol for the same time from the start of the experiment (Figure 10b), indicating a higher rate of mutual diffusion of substances in the former case. Therefore, to compare the effects of different non-solvents, it is more suitable to use the normalized length of the diffusion zone (*x*/*x*_max_).

Careful analysis of concentration profiles and interferograms reveals that the skin layer forms at an equal volume fraction of non-solvents, approximately 70% (Figure 11a). Near the interface between the skin and non-solvent, there is no jump in substances’ concentrations, indicating that the membrane skin is in equilibrium with the [EMIM]Ac/DMSO solution in non-solvent, whose content is also about 70 vol%. At the same time, the concentration change of non-solvent along the normalized length of the diffusion zone occurs differently for different non-solvents. For water and isopropanol, the concentration profiles are similar and almost symmetrical to the center of the diffusion zone, meaning that the 50% volume fraction of these non-solvents is near this center and indicating comparable diffusion rates of the [EMIM]Ac/DMSO mixture into the non-solvent and the non-solvent into the cellulose solution. In the case of methanol and ethanol, the point of their 50% volume fraction is strongly shifted towards the cellulose solution, suggesting a higher diffusion rate of the [EMIM]Ac/DMSO mixture into the non-solvent compared to the opposite process. It can be expected that a higher diffusion rate of the solvent than the non-solvent will lead to the contraction of the forming cellulose film and promote the formation of a denser membrane structure. However, a more accurate estimation of the ratio of diffusion rates can result from using the concept of the Matano plane (where an equal substance volume flow occurs in both diffusion directions) and its relative position to the borders of the forming membrane. In addition, integrating the non-solvent concentration profiles of Figure 11a allows for a precise calculation of the dependence of the interdiffusion coefficient (*D*) on the non-solvent volume fraction (see Equation (1) in Section 3.5 for a more detailed analysis).

The calculation of the mutual diffusion coefficient shows that it depends on both the nature of a non-solvent and its concentration. The highest diffusion rate is observed in the area enriched with non-solvent (Figure 11b). Penetration of any non-solvent into the cellulose solution and the formation of a skin lead to a sharp decrease in the diffusion rate. Moreover, when the diffusion rates of alcohols are analyzed separately, methanol exhibits the highest diffusion rate. This fact suggests that the precipitation of the membrane by methanol occurs faster under identical conditions, which should promote rapid cellulose coagulation and asymmetric membrane formation. The ratio between the diffusion rates of a non-solvent and solvents can be assessed by the position of the Matano plane to the skin of the forming film (indicated by vertical lines in Figure 11b). The skin formation occurs at the same volume fraction of all the non-solvents penetrating the cellulose solution, but the position of the Matano plane is different. In the case of methanol, the Matano plane passes through the center of the forming film. This position means that roughly the same number of substances enter and exit the film, i.e., the diffusion rates of the non-solvent and solvents are comparable. In this situation, one would expect no shrinkage or swelling of the forming membrane, at least because of the difference in diffusion rates of the non-solvent and solvents.

When transitioning from methanol to ethanol, the rate of mutual diffusion decreases, and the Matano plane aligns with the position of the membrane skin. In other words, more substance exits the forming membrane than enters it, i.e., the rate of solvent diffusion is higher, which is a condition for developing a denser porous structure. The lowest rate of mutual diffusion is observed in precipitation with isopropanol, but the Matano plane is located within the forming cellulose film, indicating a comparable diffusion rate of isopropanol and solvents. Likely, the rate of solvent and non-solvent interdiffusion depends not only on the viscosity of the non-solvent but also on its interaction with solvent molecules. For example, during precipitation with water, the rate of mutual diffusion of compounds is between those that appeared using methanol and ethanol. However, the Matano plane is located in the cellulose solution media, indicating a substantial water flow into the forming membrane, possibly due to the good interactions of water with DMSO and ionic liquid, which are incredibly hygroscopic compounds. The high diffusion rate of water compared to solvent molecules should lead to instantaneous cellulose precipitation and a membrane with a thick selective layer.

Thus, the nature of the used non-solvents affects the rate of mutual diffusion when obtaining a porous film and hence the rate of cellulose coagulation. Additionally, it determines the direction of substances’ mass transfer relative to the forming film, which can either shrink due to the extraction of solvent molecules and low saturation with non-solvent or expand at high diffusion rates of non-solvent molecules and low reverse flow of solvent. Let us see how this complex and varied influence of the non-solvent nature affects the morphology of cellulose membranes and their transport characteristics.

### 2.4. Morphology and Transport Properties of Cellulose Membranes

**Figure 12:**
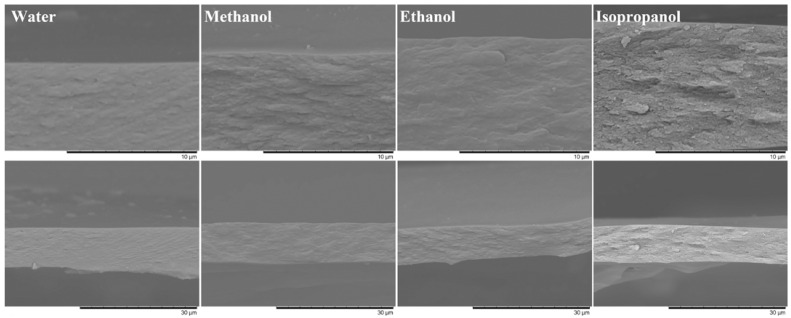
SEM images of cross-sections of cellulose membranes obtained by precipitation in different non-solvents.

The morphology of the obtained membranes is single type (Figure 12). The SEM resolution does not allow for the observation of the nanoscale pores of the membranes, but it reveals the differences in the cross-sectional roughness. When precipitated in water, the cross-sectional surface of the membrane is the smoothest, whereas precipitation in isopropanol results in the most uneven structure. In water, the diffusion rate is high, and cellulose coagulation occurs quickly, going through a gelation stage that suppresses the transformation of the microstructure of the collapsing system due to its high viscosity. As a result, evenly distributed tiny pores form, looking together like a smooth cross-sectional surface. On the contrary, isopropanol leads to a slower diffusion rate and weak structuring of the cellulose solution, which does not cause gelation, i.e., phase separation proceeds for a longer time, allowing the growth of pores and transformation of membrane structure. The greater roughness of the cross-sectional surface of the membrane obtained in isopropanol indirectly confirms its greater porosity.

The difference in porosity should lead to differences in the transport properties of cellulose films as nanofiltration membranes. These properties are the permeability of an organic liquid, such as *N*,*N*-dimethylformamide (DMF, *P*_DMF_), and the rejection (*R*) of model compounds having a relatively high molecular weight and dissolving in the filtered liquid. For such compounds, we used the dyes Orange II (350 g/mol, *R*_350_) and Remazol Brilliant Blue R (626 g/mol, *R*_626_). However, before comparing non-solvents, it is essential to compare the transport properties of membranes obtained from cellulose solutions in pure ionic liquid and its equimass mixture with DMSO using an identical non-solvent, e.g., water.

As it turns out, the use of diluted ionic liquids leads to a twofold increase in the rejection of dye molecules (from 24–31% to 55–75%, as shown in Table 1) while maintaining the permeability of the filtered liquid at 0.23–0.27 kg·m^−2^·h^−1^·atm^−1^. The unaffected permeability indicates an unchanged porosity, which means that the improvement in rejection can only be attributed to an increase in the density of the selective membrane layer (its skin). Diluted ionic liquid reduces the viscosity of the cellulose solution and the amount of a non-solvent, which can cause phase separation. The viscosity reduction should accelerate diffusion which, together with the facilitated phase separation under the influence of small amounts of non-solvent, leads to the rapid formation of the membrane skin, improving its rejection properties.

Replacement of water with alcohols as non-solvents can alter the permeability and rejection of cellulose membranes (Table 1). Methanol leads to a fivefold increase in membrane permeability, up to 1.2 kg·m^−2^·h^−1^·atm^−1^, indicating an increase in porosity likely due to a specific phase separation mechanism that does not involve gelation and a slowing down of structural reorganization. However, the increase in porosity of the membrane decreases its rejection to 35–47%, possibly due to a more porous skin layer. Ethanol yields a membrane with lower permeability (0.28 kg·m^−2^·h^−1^·atm^−1^), albeit with better rejection (56–71%). Compared to methanol, ethanol has a lower diffusion rate, while the Matano plane is located closer to the skin layer (see Figure 11b). Such a diffusion’s features should lead to the contraction of the forming film, reducing its pore size and resulting in lower permeability but better rejection. Isopropanol provides an even lower diffusion rate, a longer time for the membrane’s structural changes during phase separation, and no contraction effect due to symmetric mass transfer (since the Matano plane is inside the forming film, see Figure 11b). As a result, the resulting film has better porosity and permeability compared to using ethanol (0.42 kg·m^−2^·h^−1^·atm^−1^ versus 0.28 kg·m^−2^·h^−1^·atm^−1^) with almost no degradation in the rejection (55–68% versus 56–71%).

Thus, diluted ionic liquid improves the membrane’s rejection performance due to the accelerated formation of the skin layer upon contact with a non-solvent. In turn, alcohols, as a non-solvent alternative to water, allow for an increase in membrane permeability while maintaining its rejection performance (ethanol, isopropanol) or its slight decrease (methanol). This effect is primarily associated with the absence of gelation of the decomposing cellulose solution under the influence of the non-solvent and its resulting accelerated restructuring with the formation of pores, which is also facilitated due to the lower diffusion rate of alcohols, slowing down the phase separation.

## 3. Materials and Methods

### 3.1. Materials

Cellulose (Baikal Cellulose and Paper Mill, Baikalsk, Russia) had a polymerization degree of 600 and contained 92% alpha-cellulose and less than 6% water. [EMIM]Ac, [EMIM]Cl, and [BMIM]Ac (Sigma-Aldrich, Steinheim, Germany) were used as ionic solvents, which were diluted with DMSO (Sigma-Aldrich) as a nominal co-solvent of cellulose. Non-woven polyester fabric (Crane Technical Materials, Pittsfield, MA, USA), which had a density of 87 g/m^2^, a thickness of 90–99 μm, and an air permeability of 16.0–29.6 cm·s^−1^·Pa^−1^, was used as a micro-porous substrate for impregnating with cellulose solution and subsequent production of cellulose nanofiltration membranes. Distilled water, methanol, ethanol, and isopropanol (Himmed, Moscow, Russia) were used as non-solvents (precipitants), while hexane (Himmed) was applied for the post-processing of formed membranes. The transport properties of obtained cellulose membranes were investigated by using DMF (Sigma-Aldrich) with dissolved dyes Orange II (350 g/mol, Sigma-Aldrich) and Remazol Brilliant Blue R (626 g/mol, Sigma-Aldrich). All reagents were chemically pure and used without additional purification.

### 3.2. Preparation of Cellulose Solutions

Before dissolving cellulose as a powder, it was placed in a drying oven and kept at 80 °C for 16 h. DMSO, if used, and then ionic liquid were added to the dried powder, after which the vial with substances was sealed with polyolefin film (Parafilm M, Bemis, Neenah, WI, USA). The cellulose was dissolved under constant agitation on a magnetic stirrer C-MAG HS 7 (IKA, Staufen, Germany) at 80 °C. The DMSO mass fractions in the ionic liquid ([EMIM]Ac, [EMIM]Cl, or [BMIM]Ac) were 0, 25, 50, and 75%. The cellulose mass fraction in the IL/DMSO mixture was 14%. After the complete dissolution of cellulose, its solutions were degassed at 80 °C for 1 h, then sealed and left at 80 °C until use.

### 3.3. Formation of Membranes

A cellulose solution, preheated to 80 °C, was applied onto a nonwoven polyester substrate placed between two anti-adhesion polyester films (PPI 0501, PPI Adhesive Products, Waterford, Ireland) and then passed through hot rollers of the HLCL-1000 laminator (ChemInstruments, West Chester Township, OH, USA) at 80 °C. Immediately after, the formed film 10 × 20 cm × cm in size was immersed in a coagulation bath containing 1 L of a non-solvent at 22 ± 3 °C for 24 h. Following the coagulation process, the resultant cellulose membrane was placed in a container with 1 L of ethanol for 24 h and then in one with 1 L of hexane for 24 h. Sequential treatment with ethanol and hexane reduced the shrinkage of the cellulose layer and improved its adhesion to the nonwoven substrate, thereby eliminating layer separation during filtration experiments [90]. After being treated with hexane, all the membranes were air-dried for 24 h.

### 3.4. Rheometry of Cellulose Solutions

Rheological tests were performed using a Discovery HR-2 rotational rheometer (TA Instruments, New Castle, DE, USA) at 25 °C utilizing a cone/plate measuring module (plate diameter: 40 mm, cone/plate angle: 2°). Flow curves were obtained by stepwise increasing the shear rate from 0.001 s^−1^ to 100 s^−1^. Frequency dependences of storage and loss moduli were measured in the linear viscoelastic region by varying the angular frequency in the range of 0.0628–628 rad·s^−1^. In order to prevent phase separation of cellulose solutions due to their high hygroscopicity and possible absorption of moisture from the air, the gap between the cone and plate after placement of the cellulose solution between them was covered with silicone oil. Ordinary equations were used for calculating rheological characteristics [91], with a measurement error of no more than 5%. Each rheological curve was obtained 2–3 times to check the data reproducibility, which was always. For plotting concentration dependences, viscosity values were averaged using three flow curves.

### 3.5. Laser Interferometry

The interferometer consisted of a diffusion cell, a diode laser with 532 nm wavelength, a 3.5× objective, and a 12MP digital camera (IMX226, Sony, Tokyo, Japan). The diffusion cell represented two glass plates with a 5 nm thick gold coating applied to their surfaces. A cellulose solution was placed between these plates, separated by a wedge-shaped gap, with the following addition of a non-solvent at 22 °C. The non-solvent coming into contact with the cellulose solution formed a diffusion zone, whose interferogram was captured using the objective and camera. Interferograms were analyzed via the Matano–Boltzmann method, when the interdiffusion coefficient of cellulose solvent and non-solvent was calculated according to the following Equation:

(1) $$D=-\frac{1}{2t}\frac{dx}{d\varphi }\int_{0}^{\varphi }\left(x_{m}-x\right)d\varphi ,$$

where *t* is the time from the starting of the diffusion, *x* is the coordinate along the mass transfer axis, *x_m_* is the position of the Matano plane, and *φ* is the volume fraction of the diffusing substance (e.g., non-solvent). A detailed description of the method is provided elsewhere [89,92]. The experiment was repeated 2–3 times for each non-solvent to ensure that the diffusion zone reproduced identically.

### 3.6. Morphology and Nanofiltration Characteristics of Membranes

The morphology of cross-sectional breaks of membranes was examined on a scanning electron microscope TM3030 (Hitachi, Tokyo, Japan). Beforehand, membrane chips were obtained in liquid nitrogen and coated with a gold layer about 10 nm thick using a sputterer DSR1 (Nanostructured Coatings, Tehran, Iran).

The nanofiltration characteristics of membranes with an active area of 33.2 cm^2^ were studied at a transmembrane pressure of 20 atm in dead-end cells equipped with a magnetic stirrer [93]. The pressure in the cells was created using helium, and the amount of filtered fluid was chosen so that no more than 20% of its volume passed through the membrane during the experiment. The permeability of membranes was determined by the weight method using DMF as a test liquid:

(2) $$P=\frac{m}{APt},$$

where *m* is the mass of liquid (in kg) passing through the membrane with the area *A* (m^2^) for the time interval *t* (h) at differential pressure *P* (atm). To evaluate the nano-separation properties of the membranes, we measured their ability to reject dissolved dyes:

(3) $$R=\frac{c_{0}-c_{p}}{c_{0}}\cdot 100\%,$$

where *c*_0_ and *c*_P_ are the dye concentrations before and after filtration, respectively. At least five membranes of each type were tested to calculate the average values of the permeability and rejection. There was no noticeable adsorption of dye molecules by the membranes since they remained colorless after the experiment.

## 4. Conclusions

The novelties of this study consist of using diluted ionic liquid as a solvent for obtaining nanofiltration membranes from cellulose by a phase inversion technique and in a detailed examination of the influence of non-solvent nature on the rheological properties of a cellulose solution and the features of its non-solvent-induced phase separation. Cellulose solutions in different ionic liquids containing various nominal co-solvents representing weak or strong non-solvents of cellulose were studied comparatively for the first time, including situations when high co-solvent concentrations cause gel transition. Rheometric, interferometric, and nanofiltration investigations of the membrane-forming stages from the dilution of imidazolium ionic liquids ([EMIM]Ac, [EMIM]Cl, and [BMIM]Ac) with DMSO to the production of cellulose membranes by the action of various non-solvents (water, methanol, ethanol, and isopropanol) revealed the following:

1. Any low-viscosity liquids that are not solvents of cellulose nevertheless can be its nominal co-solvents at low doses, reducing the viscosity of the forming solution up to 50 times (the case of [BMIM]Ac/DMSO = 25/75) by lowering the viscosity of the used ionic liquid and deteriorating its thermodynamic affinity for cellulose macromolecules. This effect is observed for ionic liquids that are amorphous at the test temperature ([EMIM]Ac and [BMIM]Ac) but is inverse for the crystallizing ionic liquid ([EMIM]Cl), whose dilution increases the cellulose solution viscosity.
2. Moderate dilution of the amorphous ionic liquid with a non-solvent causes strong (in the case of water) or weak (alcohols) structuring of the cellulose solution, manifesting as gel formation or an anomaly of low-frequency storage modulus, respectively. In the row of water, methanol, ethanol, isopropanol, and DMSO, the characteristic mass fraction of the non-solvent that causes the structuring increases, and DMSO can cause both strong and weak structuring, depending on its concentration and type of ionic liquid.
3. The transition from water and methanol to ethanol and then isopropanol reduces the rate of interdiffusion of substances during the phase separation of the cellulose solution up to four times and thus slows it down. Moreover, a change in the used non-solvent can shift the mass transfer direction relative to the forming cellulose membrane, contributing to its contraction (ethanol) or swelling (water).
4. The transition to diluted ionic liquid ([EMIM]Ac/DMSO equimixture in our case) enhances the rejection properties of cellulose nanofiltration membrane without changing its permeability performance. This effect is possibly due to the accelerated formation of the membrane skin layer because of the decreased viscosity of the cellulose solution (and consequently accelerated diffusion of the non-solvent) and reduced thermodynamic quality of the solvent, resulting in phase separation under the action of less quantity of the non-solvent. As a result, the membrane skin becomes denser and finely porous, improving rejection without changing the porosity of the support layer, i.e., maintaining high permeability. In turn, the replacement of water by alcohols allows for increasing permeability of the cellulose membrane while maintaining (ethanol and isopropanol) or slightly decreasing (methanol) its rejection properties. This change occurs probably due to better pore growth in the membrane support layer thanks to the absence of the gelation stage during phase separation and its slowdown. The resulting support layer becomes less dense and more porous, raising permeability, while the skin’s density decreases only slightly or remains unchanged, maintaining good rejection.

Future research should investigate more concentrated cellulose solutions in highly diluted ionic liquids, including those containing a hard non-solvent, to evaluate their use in improving the nanofiltration characteristics of cellulose membranes.

## Figures and Tables

**Table 1:** Nanofiltration characteristics of cellulose membranes.

Solvent	Non-Solvent	*P*_DMF_, kg·m^−2^·h^−1^·atm^−1^	*R*_350_, %	*R*_626_, %
[EMIM]Ac	water	0.27 ± 0.05	24 ± 4	31 ± 5
[EMIM]Ac/DMSO	water	0.23 ± 0.05	55 ± 7	75 ± 5
[EMIM]Ac/DMSO	MeOH	1.20 ± 0.20	35 ± 4	47 ± 9
[EMIM]Ac/DMSO	EtOH	0.28 ± 0.09	56 ± 7	71 ± 9
[EMIM]Ac/DMSO	iPrOH	0.42 ± 0.24	55 ± 9	68 ± 10

## Data Availability

The data presented in this study are available upon request from the corresponding author.
